# Primary tuberculosis of the parotid gland: A forgotten diagnosis about a case!

**DOI:** 10.1002/ccr3.3954

**Published:** 2021-05-04

**Authors:** Elmostafa Benaissa, Mohamed Habib Bahalou, Yassine Safi, Fatna Bssaibis, Yasssine Benlahlou, Mariama Chadli, Adil Maleb, Mostafa Elouennass

**Affiliations:** ^1^ Epidemiology and Bacterial Resistance Research Team/BIO‐INOVA Centre Faculty of Medicine and Pharmacy (University Mohammed V) Rabat Morocco; ^2^ Department of Bacteriology Mohammed V Military Teaching Hospital/Faculty of Medicine and Pharmacy (University Mohammed V) Rabat Morocco; ^3^ ORL and Maxillofacial Surgery Department Mohammed V Rabat Military Hospital Rabat Morocco; ^4^ Laboratory of Microbiology Mohammed VI University Hospital/Faculty of Medicine and Pharmacy (University Mohammed the first) Oujda Morocco

**Keywords:** histological study, parotid gland, PCR, tuberculosis, Ziehl‐Nielsen stain

## Abstract

The diagnosis of tuberculosis must be made in the face of any cervical swelling, and the treatment is essentially medical.

## Graphical abstract

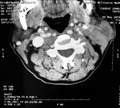

## 1 INTRODUCTION

Primary tuberculosis of the parotid gland is rare even in endemic countries. The clinical manifestations are polymorphic and nonspecific. It is important for clinicians to be aware of this pathology.

Tuberculosis is a granulomatous infection caused by *Mycobacterium tuberculosis* or *bovis* that can affect all organs. In the ENT area, lymph node involvement is the most common. Isolated localization of tuberculosis in the salivary glands, especially the parotid gland, is extremely rare.^1^, ^2^ The clinical presentation is polymorphic and nonspecific.

We report a case of primary tuberculosis of the left parotid gland simulating a parotid tumor in a 50‐year‐old woman, and we discuss the different diagnostic and therapeutic elements of this pathology, insisting on the need to demand a molecular test (PCR) combined with a histological study.

## 2 CASE REPORT

A 50‐year‐old woman, without any notable pathological antecedent, without any notion of tuberculosis contagion, and consuming unpasteurized raw milk, consulted for cervical tumefaction. The history of the disease goes back to 8 months with the appearance of a left cervical swelling progressively increasing in volume, which presented a month ago a redness with pain. In addition, the patient complains of chills and night sweats and unquantified weight loss.

The clinical examination finds a left cervical mass located in the retromandibular and under the auricle of the left ear painful with a red facing skin measuring 3*3 cm hard and adherent, without peripheral facial paralysis and without inflammation of the ositum of the stenon. The rest of the ENT, lymph node, and lung examination is without particularities.

Cervical ultrasonography showed a heterogeneous hypoechoic parotid gland process containing areas of necrosis.

**FIGURE 1:**
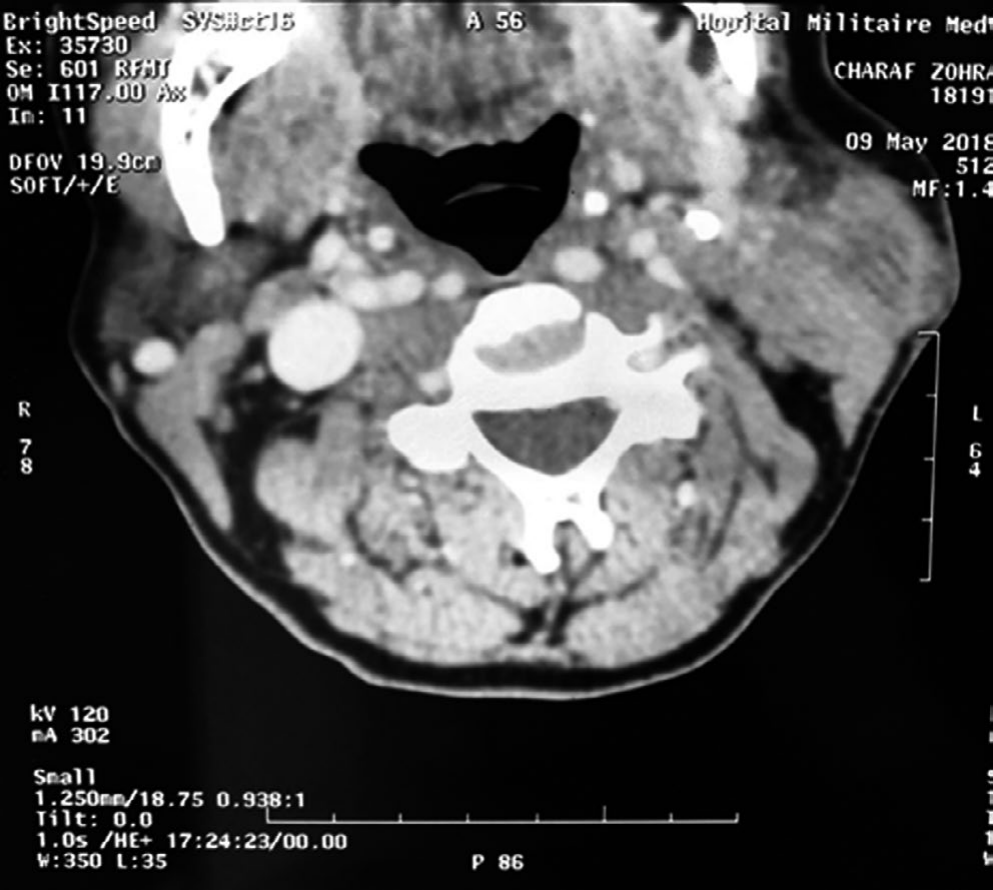
Axial section in parenchymal window showing a left intraparotid collection

Cervical CT scan showed the presence of a very limited, hypodense, bilobed formation at the lower pole of the left parotid gland, which rises in the periphery after injection of contrast material, coming into intimate contact with the sternocleidomastoid muscle and measuring 31 × 26 mm (Figure 1).

**FIGURE 2:**
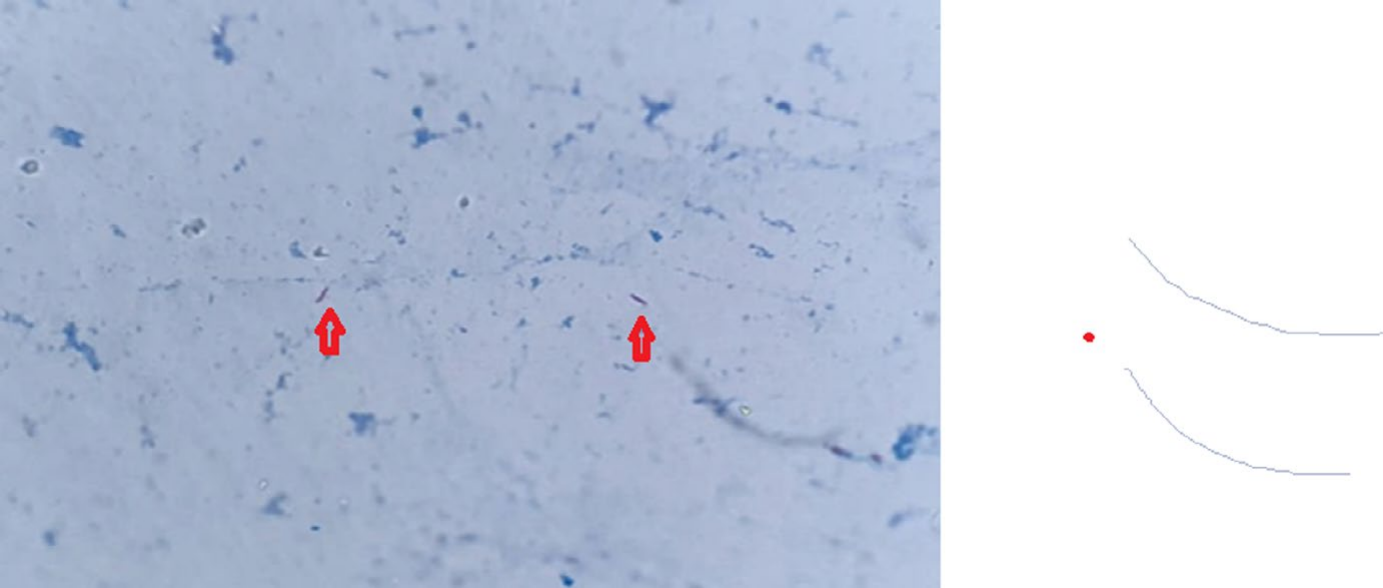
Ziehl‐Nielsen stain that shows acid‐fast bacilli (100×)

The right parotid gland was without notable abnormalities with no cervical adenopathies. The biological assessment was normal, as was the chest X‐ray. During the explorations, the evolution was marked by spontaneous fistulization of the mass at the cervical level, with pus outcome. A biopsy curettage through the fistulous orifice was performed, with bacteriological and anatomopathological samples. The bacteriological study included a molecular study using GeneXpert MTB/RIF (Cepheid) which allowed the detection of *Mycobacterium tuberculosis* complex with absence of rifampicin resistance in <2 hours and also a microscopic examination after ziehl Neelsen staining which was positive (Figure 2) and a culture on solid medium type Lowenstein‐Jensen^®^(LJ) and on liquid medium type MGIT^®^ (Mycobacteria Growth Indicator Tube) which were positive respectively 21 days and 12 days. Therapeutic treatment was initiated based on molecular data.

The histopathological study confirmed the molecular diagnosis by showing epithelioid and gigantocellular granulomas with caseous necrosis, with no histological sign of malignancy. Hence, the interest to request a molecular test associated with an anatomopathological study.

An extended (9‐month) four‐drug regimen (H: isoniazid, E: ethambutol, R: rifampin, and Z: pyrazinamide) was initiated according to our usual protocol: 2 RHZE/7 RH. After 6 years, the evolution was satisfactory without any relapse.

## 3 DISCUSSION

First described in 1894 by von Stubenrauch, parotid tuberculosis is rare. More than 90% of described cases occur in developing countries.^3^ It most often affects young people between 20 and 40 years of age. However, our patient was 50 years old.

Parenchymatous parotid localization is most often seen in disseminated tuberculosis, the diffusion being by hematogenous or lymphatic route from a pulmonary focus or isolated, posing a problem of differential diagnosis with the tumor pathology of the parotid gland. In this case, the Mycobacterium, localized at the oropharyngeal level, passes through a retrograde ductal route through the stenon duct. It must be distinguished from a tubercular involvement of the intraparotid nodes, which is much more frequent.

Clinically, parotid tuberculosis usually manifests itself as a unilateral parotid swelling of progressive onset, which may be diffuse or nodular, resulting in a pseudotumoral syndrome. On the other hand, the presence of a cutaneous fistula is very suggestive of an inflammatory pathology. General signs of tubercular impregnation are rarely present, but must be sought and may guide the diagnosis, as well as the patient's geographical origin.^3^

Radiological examination data (ultrasound, CT scan, and MRI) generally do not allow the diagnosis to be made. A chest X‐ray should always be requested in order to look for a possible primary focus. Biologically, an inflammatory syndrome is usually present, and the tuberculin intradermal reaction is not always positive.^2^, ^3^, ^4^, ^5^

Thus, there is no clinical, radiological, or biological evidence to support a diagnosis of parotid tuberculosis. Therefore, the differential diagnosis of the diffuse forms is mainly based on lithiasis parotiditis or carcinoma, while the circumscribed form is more suggestive of a cyst, adenitis, or mixed tumor.

The challenge for the practitioner is, therefore, to make the diagnosis before the surgical exploration and histological examination of the parotidectomy specimen, especially if this picture is seen in the child who is exceptionally affected and in whom the diagnosis is, therefore, very rarely mentioned.

Fine needle cytopuncture with culture of the puncture fluid may be useful, but is only of value if it is positive. It has a specificity of 81% and a sensitivity of 94%.^6^ Currently, most authors recommend PCR (polymerase chain reaction) gene amplification techniques after glandular cell culture to increase the positive cytopuncture results.^6^ In the case of our patient, molecular diagnosis was able to highlight the Mycobacterium tuberculosis complex. Others propose a therapeutic test for tuberculosis patients when the diagnosis is strongly suspected.^3^, ^5^ However, in some cases, only histological examination of satellite lymphadenopathy or sometimes even parotidectomy will allow the diagnosis to be made by highlighting the classic epitheliogigantocellular granuloma with caseous necrosis.^4^, ^6^

Exofacial parotidectomy has long been recommended for diagnostic purposes and to allow better diffusion of antituberculosis drugs. It is currently abandoned by all the authors because of its non‐negligible morbidity and the effectiveness of medical treatment alone. It is based on the prescription of long‐term antituberculosis drugs, generally for 6‐9 months, allowing a rapid disappearance of the parotid tumor syndrome.^3^, ^5^, ^6^

## 4 CONCLUSION

If lymph node localizations are excluded, ENT tuberculosis is a rare condition. It can affect all age groups, although it is more common in young adults. The clinical and paraclinical signs are not very specific; the diagnosis is essentially based on histology and bacteriology, hence, the importance of multiplying biopsy samples if necessary. In doubtful cases, PCR has proven its effectiveness.

In an endemic country such as Morocco, tuberculosis should always be mentioned as a differential diagnosis. The evolution under well‐conducted medical treatment is excellent.

## CONFLICT OF INTERESTS

The authors declare no competing interest.

## AUTHOR CONTRIBUTIONS

BE, BHM, and SY: involved in drafting of manuscript. BY, MA, CM, and BF: involved in revising of manuscript. ELM: provided final approval of version to be published. All authors approved the final version of the manuscript and agree to be accountable for all aspects of the work ensuring that questions related to the accuracy or integrity of any part of the work are appropriately investigated and resolved.

## ETHICAL APPROVAL

Verbal and written informed consent was obtained from the patient to participate in this study.

## Data Availability

The data used and/or analyzed during the current study are available from the corresponding author on reasonable request.
